# Whole brain radiotherapy combined with intrathecal liposomal cytarabine for leptomeningeal metastasis—a safety analysis and validation of the EANO-ESMO classification

**DOI:** 10.1007/s00066-022-01910-9

**Published:** 2022-03-10

**Authors:** Sarah Iglseder, Martha Nowosielski, Gabriel Bsteh, Armin Muigg, Johanna Heugenhauser, Elke Mayer, Astrid Grams, Günther Stockhammer, Meinhard Nevinny-Stickel

**Affiliations:** 1 Department of Neurology, Medical University of Innsbruck, Anichstraße 35, 6020 Innsbruck, Austria; 2 Department of Neurology, Medical University of Vienna, Vienna, Austria; 3 Department of Therapeutic Radiology and Oncology, Medical University of Innsbruck, Innsbruck, Austria; 4 Department of Neuroradiology, Medical University of Innsbruck, Innsbruck, Austria

**Keywords:** Leptomeningeal metastases, Intrathecal therapy (DepoCyte®), Survival analysis, Outcome

## Abstract

**Background:**

Although there is no proven standard therapy for leptomeningeal metastases (LM), treatment often includes intrathecal chemotherapy combined with whole brain radiation therapy (WBRT). Little is known about the toxicity of such combination therapies. We performed a retrospective safety analysis for the combination of intrathecal liposomal cytarabine with WBRT in patients with LM and validated the EANO-ESMO (European Association of Neuro-oncology—European Society for Medical Oncology) classification in this unique cohort.

**Methods:**

Treatment toxicities in patients diagnosed with LM between 2004 and 2014 were retrospectively analyzed according to RTOG (Radiation Therapy Oncology Group) toxicity criteria and NCI CTCAE V5.0 (National Cancer Institute Common Terminology Criteria for Adverse Events, Version 5.0). Diagnostic criteria and treatment response as assessed by EANO-ESMO classification were correlated with survival by Kaplan–Meier analysis and Breslow test.

**Results:**

In all, 40 patients with LM who were treated with combined WBRT and intrathecal cytarabine, were identified. Ten patients (25%) experienced adverse events ≥grade 3 according to RTOG toxicity criteria; in 22 patients (55%) NCI CTCAE ≥grade 3 were detected. Median overall survival was 124 days. Median time to neurological progression was 52 days. Patients with positive cerebrospinal fluid (CSF) cytology (*n* = 26) showed worse prognosis compared to patients with negative CSF cytology (*n* = 14; mOS (median overall survival) 84 days versus 198 days, *p* = 0.006, respectively). The EANO-ESMO response assessment was significantly associated with survival: “stable” (*n* = 7) mOS 233 days, “response” (*n* = 10) mOS 206 days, “progression” (*n* = 17) mOS 45 days, “suspicion of progression” (*n* = 6) mOS 133 days; overall, *p* < 0.001.

**Conclusions:**

In this retrospective analysis, combined treatment of WBRT and intrathecal liposomal cytarabine shows an acceptable safety profile and may indicate a trend towards improved efficacy. The EANO-ESMO classification for diagnosis and treatment response predicts survival.

**Supplementary Information:**

The online version of this article (10.1007/s00066-022-01910-9) contains supplementary material, which is available to authorized users.

## Introduction

Leptomeningeal metastasis (LM) is a rare but devastating neurologic manifestation of systemic cancer, affecting patients with lymphoproliferative malignancies and solid tumors, mainly from lung and breast cancer as well as melanoma [1–3]. Early diagnosis and immediate initiation of therapy [4] is important to extend overall survival and to maintain quality of life [5, 6]. Whole brain radiation therapy (WBRT) is the most widely used treatment for patients with disseminated brain metastases and bulky meningeal disease [7, 8]. Bodensohn et al. demonstrated that a simultaneous stereotactic radiosurgery using a single-isocenter dynamic conformal arc (SIDCA) seems to be a feasible and safe treatment for patients with multiple brain metastases [9]. However, WBRT is the conventional treatment for patients with LM and provides attenuation of prevalent neurological signs and symptoms [10]. When comparing quality of life data of patients with brain metastasis who underwent WBRT or stereotactic radiotherapy, the results showed significant differences in symptom scores between the two radiotherapy techniques [11].

Although, no randomized trial has demonstrated that intrathecal chemotherapy prolongs survival in LM patients, intra-cerebrospinal fluid (CSF) chemotherapy is commonly used to treat LM across Europe [12]. Liposomal cytarabine offers the advantage of extended CSF half-life compared to other intrathecally applicable agents resulting in cytotoxic concentrations of at least 14 days and was approved for the treatment of lymphomatous meningitis [13]. It is also used off-label for LM from solid tumors as it has shown to increase neurological progression-free survival [13].

Knowledge on toxicities and efficacy of combined CNS-directed radiotherapy and intra-CSF chemotherapy is limited [14, 15]. So far, no study systematically evaluated the toxicity of liposomal cytarabine in combination with WBRT. Even though in summer 2018, the European Commission withdrew the marketing authorization for liposomal cytarabine (DepoCyte®, Pacira Limited, Bourne End, United Kingdom) in the European Union at the request of the marketing authorization holder for commercial reasons, we performed a retrospective analysis of patients treated for LM in our institution by a combination therapy of WBRT and liposomal cytarabine, given either concomitantly or sequentially. Furthermore, we used the opportunity of this unique patient cohort to evaluate the recently proposed European Association of Neuro-oncology—European Society for Medical Oncology (EANO-ESMO) classification on diagnostic criteria and treatment response.

## Patients and methods

All patients seen in the departments of Neurology and Therapeutic Radiology and Oncology between 2004 and 2014 with LM from solid, lymphoproliferative or hematological malignancies as defined by the EANO-ESMO criteria (see definition below) and treated with combined WBRT and liposomal cytarabine were included. Between October 2010 and August 2012, our center was part of a multicenter study investigating toxicity and feasibility of combined WBRT and liposomal cytarabine in the treatment of LM (NCT00854867). The clinical trial NCT00854867 was conducted in accordance with national and local laws and was approved by the local ethical review committee (UN3719-LEK). All subjects and/or their guardians/legally authorized representatives were provided with oral and written information describing the nature and duration of the study, its purpose, the procedures to be performed, the potential risks and benefits involved, and any potential discomfort. Due to poor recruitment, however, the study was closed after enrolling 18 patients in seven centers within 3 years. All other patients who have been diagnosed with leptomeningeal metastasis by positive lumbar CSF cytology and/or magnetic resonance imaging (MRI) followed by WBRT combined with concomitant or sequential DepoCyte® were retrospectively identified. This retrospective analysis was approved by the local ethical review committee (UN5203). In this retrospective analysis, we also included patients who formally were enrolled in this prospective study as well. We identified patients who were treated concomitantly with WBRT and liposomal cytarabine (first dose of liposomal cytarabine was administered within the first week of WBRT) and patients who received liposomal cytarabine directly after completion of WBRT. Therefore, we defined a concomitant group and a sequential group for further analysis in order to detect any differences in toxicities and treatment responses. We excluded patients with previous WBRT and previous intrathecal treatment.

### Diagnostic criteria

The diagnosis of LM was based on the EANO-ESMO criteria [15]. “LM confirmed” required the presence of tumor cells in the CSF, “LM probable” was defined as showing typical neuroimaging findings assessed by MRI such as leptomeningeal enhancement of the brain, spinal cord, cauda equina or subependymal areas with extension into the sulci of the cerebrum or folia of the cerebellum or both [16] with typical neurological clinical signs. “LM possible” showed typical MRI findings as described above without clinical signs. Patients were furthermore categorized as type I or type II LM. Type I required a positive biopsy or CSF cytology, while type II was defined as negative or unequivocal CSF or biopsy. Subcategories of these two principal types include different MRI patterns. Type A shows a linear, type B a nodular, type C a combination of a linear and nodular pattern and type D represents a normal MRI [15].

### Documentation of adverse events

Adverse events (AEs) were assessed by reviewing medical reports and laboratory results according RTOG (Radiation Therapy Oncology Group) [17] and NCI CTCAE (National Cancer Institute Common Toxicity Criteria Adverse Events Version 5.0) V5.0 toxicity criteria [18]. Identification of episodes of drug-related arachnoiditis were based on a standardized algorithm. Patients were scored if, within 4 days of drug injection, they developed either: neck rigidity, neck pain, or meningismus, or if they developed any two of the following signs or symptoms at the same time: nausea, vomiting, headache, fever, back pain, or aseptic CSF pleocytosis.

### Response to treatment

Imaging response assessment based on MRI scans were performed every 3 months. Response to treatment was defined according to the EANO-ESMO classification 8–12 weeks after start of treatment [15]. “EANO-ESMO response” was defined as clinically improved or stable, neuroimaging has improved, CSF cytology has improved or was stable. “EANO-ESMO stable” was interpreted as clinically stable, neuroimaging and CSF cytology were stable. “Suspicion of progression” represented a clinical deterioration, a stable neuroimaging, a stable CSF cytology or the patients became clinically stable or worse, neuroimaging was stable and CSF cytology got worse (increased tumor cell counts). “Progression” was defined as worsening of neuroimaging or worse or de novo positive CSF cytology. Time to neurological progression (TTNP) was defined as the time from diagnosis of LM to neurological progression secondary to LM. Overall survival (OS) was defined as the time from LM diagnosis until death.

### Statistical analysis

All described results are reported as median with range or 95% confidence interval (CI) for continuous variables and as frequencies or percentages for categorical variables. Toxicities were descriptively reported and their association with systemic therapy and application form were analyzed by cross-table analysis and Fisher’s exact test. At the time of statistical evaluation (February 2021) all patients had died. Kaplan–Meier curves were plotted to estimate the association of diagnostic types and response to treatment using Breslow test. For the following clinically relevant variables, univariate survival subgroup analyses were conducted: median age at initial diagnosis, median KPS (Karnofsky performance status), and number of extraneuronal metastatic sites. All statistical tests were performed using SPSS 26.0 (IBM, Armonk, NY, USA).

## Results

### Patient characteristics

Between October 2004 and May 2014, a total of 40 patients diagnosed with LM were treated with WBRT and intrathecal liposomal cytarabine, either given concomitantly (*n* = 31, 75%) or sequentially (*n* = 9, 25%). DepoCyte®, either given concomitant or sequential to WBRT, was intrathecally administered at a dose of 50 mg every 2 weeks for a total of four treatments and then once every 4 weeks in responding or stable patients. A total of 189 cycles of intrathecal DepoCyte® were administered with a median of five (range 1–15) in the concomitant and three (range 1–13) in the sequential group.

Demographic as well as disease-related variables of the two therapeutic groups are summarized in Table 1. According to the EANO-ESMO classification, in 26 patients (65%) LM was confirmed (=type I) and in 14 patients (35%) LM was probable (type II), while no patient was classified as “LM possible”. Thirteen patients showed a linear MRI pattern (type A, 32.5%), 5 patients showed a nodular pattern (type B, 12.5%), 15 patients showed a linear + nodular MRI pattern (37.5%) and 7 patients had a normal MRI (17.5%; Table 2).

**Table 1:** Patient characteristics

	Overall, *n* = 40	Concomitant, *n* = 31	Sequential, *n* = 9
*Age (years)*^*a*^	59 (38–80)	59 (38–80)	59 (44–73)
*KPS (%) at diagnosis of LM*^*a*^	70 (50–100)	70 (60–100)	70 (50–90)
KPS ≥60%^b^	31 (77.5)	24 (77.4)	7 (77.8)
KPS <60%^b^	9 (22.5)	7 (17.5)	2 (22.2)
*Sex*			
Male^b^	10 (25.0)	7 (22.6)	3 (33.3)
Female^b^	30 (75.0)	24 (77.4)	6 (66.7)
*Primary tumor*			
NSCLC^b^	15 (37.5)	10 (32.3)	5 (55.6)
Breast^b^	12 (30.0)	9 (29.0)	3 (33.3)
Non solid^b,c^	3 (7.5)	3 (9.7)	0 (0.0)
Others^b,d^	10 (25.0)	9 (29.0)	1 (11.1)
*Brain metastases*^*b*^	21 (52.5)	18 (58.1)	3 (33.3)
*Extraneuronal metastases (n)*			
0^b^	8 (20.0)	6 (19.4)	2 (22.2)
1–3^b^	6 (15.0)	5 (16.1)	1 (11.1)
>3^b^	26 (65.0)	20 (64.5)	6 (66.6)
*Concomitant systemic pharmacotherapy*^*b,e*^	20 (50.0)	18 (58.0)	2 (22.2)
*Previous cranial or spinal radiotherapy*^*b*^	4 (10.0)	2 (6.5)	2 (22.2)
*Presenting symptoms of LM*			
Headache^b^	8 (20.0)	6 (19.4)	2 (22.2)
Cranial nerve dysfunction^b^	13 (32.5)	10 (32.3)	3 (33.4)
Spinal cord dysfunction^b^	2 (5.0)	1 (3.2)	1 (11.1)
Peripheral motor/sensory NP^b^	7 (17.5)	5 (16.1)	2 (22.2)
Others^b,f^	10 (25.0)	9 (29.0)	1 (11.1)

*CSF* cerebrospinal fluid, *KPS* Karnofsky performance status, *LM* leptomeningeal metastases, *MRI* magnetic resonance imaging, *n* number, *NP* Neuropathy, *NSCLC* non-small lung cancer

^a^Median (range)

^b^Absolute number (percentage)

^c^Non-solid: non-Hodgkin lymphoma, multiple myeloma

^d^Others: colorectal cancer, gastric cancer, esophageal cancer, uterine cancer, ovarian cancer, rhabdomyosarcoma, glioblastoma, malignant melanoma

^e^Concomitant systemic pharmacotherapy: Vemurafenib, Temozolomide, R‑Benda, Bevacizumab, Cisplatin, Abraxane, Taxotere, Tamoxifen, Capecitabine, FOLFOX, Mycocet, Epirubicin, Erlotinib

^f^Others: epilepic seizure, vertigo, ataxia, aphasia

**Table 2:** Diagnostic criteria as assessed by the EANO-ESMO classification [15]

		Overall (*n* = 40)	Concomitant (*n* = 31)	Sequential (*n* = 9)
*Type I*^*a*^	IA	8 (20.0)	7 (22.6)	1 (11.1)
	IB	1 (2.5)	1 (3.3)	0 (0.0)
	IC	11 (44.0)	8 (25.8)	3 (33.3)
	ID	6 (15)	4 (12.9)	2 (22.2)
*Type II*^*a*^	IIA	5 (12.5)	4 (12.9)	1 (11.1)
	IIB	4 (10.0)	3 (9.7)	1 (11.1)
	IIC	4 (10.0)	3 (9.7)	1 (11.1)
	IID	1 (2.5)	1 (3.2)	0 (0.0)
*LM confirmed*^*a*^		26 (65.0)	20 (64.5)	6 (66.7)
*LM probable*^*a*^		14 (35.0)	11 (35.5)	3 (33.3)
*LM possible*^*a*^		0 (0.0)	0 (0.0)	0 (0.0)

*EANO-ESMO* European Association of Neuro-oncology—European Society for Medical Oncology, *LM* leptomeningeal metastases, *n* number

^a^Absolute number (percentage)

### Therapy

Median overall duration of WBRT was 28 days (range 3–36 days). A median total dose of 38.4 Gy (range 7.8–40 Gy) was applied. Patients received 3 Gy WBRT on days 1 and 2 followed by 1.8 Gy per day, 5 days a week. In 6 patients (15%) radiotherapy had to be terminated prematurely (in 4 patients due to disease progression, in 2 because of patient’s wish).

Liposomal cytarabine was administered via intralumbal route in all patients at a dose of 50 mg every 2 weeks [13, 19]. In the concomitant group liposomal cytarabine was given on day 3–5 during the first week of WBRT and in the sequential group on day 29–31. All patients received dexamethasone to decrease the risk of drug-related arachnoiditis. Concurrent organ-specific systemic pharmacotherapy was administered in 18 patients (45%; Table 1).

### Toxicity RTOG

Acute adverse events (AEs) of any grade according to RTOG toxicity criteria were detected in 24 patients (60%). Ten patients (25%) experienced 13 acute AEs grade 3 or higher (Table 3). No significant differences in RTOG toxicities were observed between the two application groups. Headache was most prominent (*n* = 5, 12.5%), followed by dermatitis (*n* = 3, 7.5%), visual field restriction (*n* = 2, 5%), hematological toxicities (*n* = 2, 5%) and external otitis (*n* = 2, 5%).

**Table 3:** WBRT^b^ associated toxicity ≥grade 3 (RTOG toxicity criteria) [17]

Symptoms^a^	Overall (*n* = 40)	Concomitant (*n* = 31)	Sequential (*n* = 9)
Dermatitis	3 (7.5)	2 (6.5)	1 (11.1)
Visual field restriction	2 (5.0)	2 (6.5)	0 (0.0)
External otitis	1 (2.5)	1 (3.2)	0 (0.0)
Headache	5 (12.5)	4 (12.9)	1 (11.1)
Hematological toxicity^b^	2 (5.0)	1 (3.2)	1 (11.1)

*n* number, *RTOG* Radiation Therapy Oncology Group, *WBC* white blood count, *WBRT* whole brain radiotherapy

^a^Absolute number (percentage)

^b^Grade 3: WBC (G/l) 1.0–<2.0, Platelets (G/l) 25–<50, Neutrophils (G/l) 0.5–<1.0, Hemoglobin (G/l) 50–<100; Grade 4: WBC (G/l) <1.0, Platelets (G/l) <25 or spontaneous bleeding, Neutrophils (G/l) <0.5 or sepsis

### Toxicity CTCAE

Acute AEs of any grade according to NCI CTCAE toxicity criteria were detected in 27 patients (67.5%), while 13 patients had no toxicity at all (32.5%). Thirty-six chemotherapy induced AEs ≥grade 3 were detected in 22 patients (55%) and are summarized in Table 4. Cognitive disturbance (*n* = 8, 20%) and cranial nerve dysfunction (*n* = 8, 20%) were followed by headache (*n* = 7, 17.5%), nausea/vomiting (*n* = 5, 12.5%), somnolence (*n* = 5, 12.5%), hematological toxicities (*n* = 4, 10%) and other CNS disorders (*n* = 4, 10%). Two patients (5%) experienced a drug-related arachnoiditis. Again, no significant differences in the frequency of toxicity were found between the two application groups. Concurrent systemic therapy was not associated with an increased frequency of toxicities (Supplement Table 1).

**Table 4:** DepoCyte® induced toxicity ≥grade 3 (NCI CTCAE^b^ V5.0) [18]

Symptoms^a^	Overall (*n* = 40)	Concomitant (*n* = 31)	Sequential (*n* = 9)
Headache	7 (17.5)	6 (19.4)	1 (11.1)
Cognitive disturbance	8 (20.0)	5 (16.1)	3 (33.3)
Ataxia	1 (2.5)	0 (0.0)	1 (11.1)
Somnolence	5 (12.5)	3 (9.7)	2 (22.2)
Cranial nerve dysfunction	8 (20.0)	4 (12.9)	4 (44.4)
Peripheral motor/sensory neuropathy	2 (5.0)	1 (3.2)	1 (11.1)
Hematological toxicity^b^	4 (10.0)	4 (12.9)	0 (0.0)
Other^c^ CNS disorder	4 (10.0)	3 (9.7)	1 (11.1)
Nausea/vomiting	5 (12.5)	4 (12.9)	1 (11.1)
Conus/cauda syndrome	0 (0.0)	0 (0.0)	0 (0.0)
Drug-related meningitis	2 (5.0)	1 (3.2)	1 (11.1)

*n* number, *NCI CTCAE* National Cancer Institute Common Toxicity Criteria Adverse Events, *CNS* central nervous system, *WBC* white blood count

^a^Absolute number (percentage)

^b^Grade 3: WBC (G/l) 1.0–<2.0, Platelets (G/l) 25–<50, Neutrophils (G/l) 0.5–<1.0, Hemoglobin (G/l) <80, transfusion indicated; grade 4: WBC (G/l) <1.0, Platelets (G/l) <25, Neutrophils (G/l) <0.5, Hemoglobin Life-threatening consequences; urgent intervention indicated

^c^Severe, medically significant or life-threatening consequences which require hospitalization, prolongation of existing hospitalization or urgent interventions

### Survival and response

Median overall survival (mOS) was 124 days [CI 72.9; 175.1]. No difference in survival between the different application forms could be detected (concomitant mOS 124 days [CI 79.2; 168.7], sequential mOS 122.2 days [CI 0; 366.6], *p* = 0.702). Median TTNP was 52 days [CI 41.1; 62.8], in the concomitant group 52 days [CI 39.7; 64.7] and 54 days [CI 0; 144.7] in the sequential group, respectively.

**Fig. 1:**
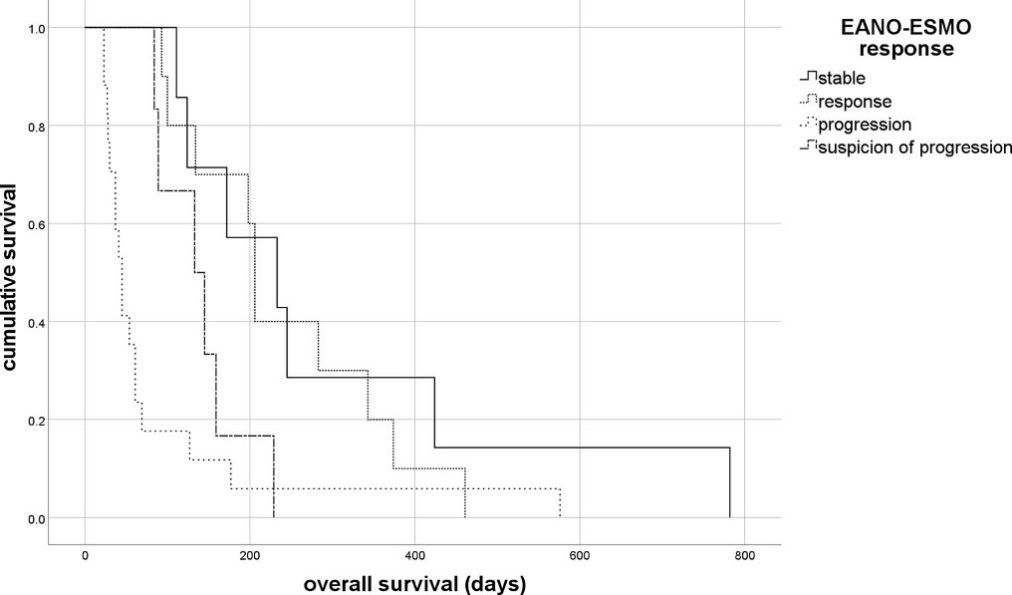
Kaplan–Meier curves and EANO-ESMO response assessment. The EANO-ESMO response assessment correlated significantly with survival (“stable” [*n* = 7]: median overall survival, mOS, 233.0 days [confidence interval, CI 76.5; 389.5]; “response” [*n* = 10]: mOS 206.0 days [CI 193.9; 218.9]; “progression” [*n* = 17]: mOS 45.0 days [CI 34.4; 55.6]; “suspicion of progression” [*n* = 6]: mOS 133.0 days [CI 65.8; 200.2]; overall: *p* < 0.001). There was no significant difference between the “stable” and “response” group; *p* = 0.773

The EANO-ESMO response assessment was significantly associated with survival. ESMO stable (*n* = 7) showed a mOS of 233 days [CI 76.5; 389.5], ESMO responses (*n* = 10) a mOS of 206 days [CI 193.9; 218.9], ESMO progression (*n* = 17) a mOS of 45 days [CI 34.4; 55.6], and suspicion of progression (*n* = 6) a mOS of 133 days [CI 65.8; 200.2] (overall *p* < 0.001, Fig. 1). Furthermore, there was no significant difference between the ESMO stable and response group (*p* = 0.773). No significant differences between the four ESMO responses or applied therapy (concomitant versus sequential) could be detected (*p* = 0.412, Supplement Table 2). No other clinically relevant parameters were significantly associated with survival in univariate analysis (median age *p* = 0.561; KPS *p* = 0.765; number of extraneuronal metastatic sites *p* = 0.192). Interestingly, there was a difference in EANO-ESMO responses and frequency of toxicities according to RTOG criteria (*p* = 0.045, Table 5).

**Table 5:** Toxicity and EANO-ESMO response [15]

	RTOG toxicity occurrence		*p*-value
EANO-ESMO response	No	Yes	
Stable	1	6	0.045^a^
Response	2	8	**–**
Progression	6	9	**–**
Suspicion of progression	5	1	**–**

*EANO-ESMO* European Association of Neuro-oncology—European Society for Medical Oncology, *RTOG* Radiation Therapy Oncology Group

^a^Fisher’s exact test

**Fig. 2:**
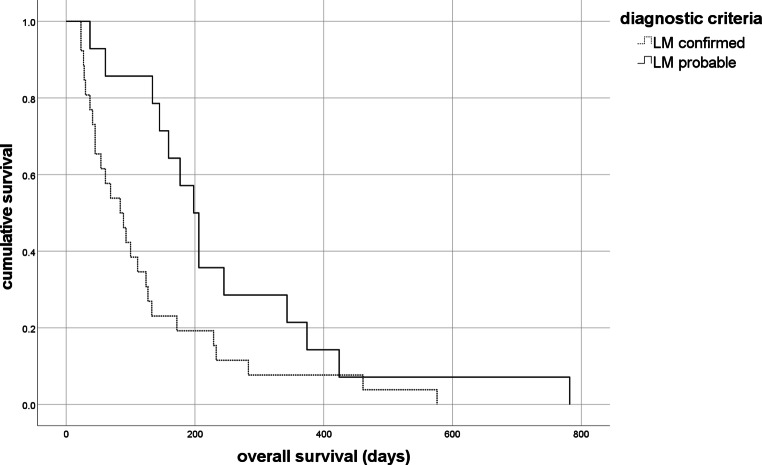
Kaplan–Meier curves and diagnostic criteria. Type I (*n* = 26) showed a worse prognosis compared to type II (*n* = 14; median overall survival, mOS, 84 days [confidence interval, CI 44.0; 124.0] versus 198.0 days [CI 162.6; 233.4], *p* = 0.006, Breslow test)

When comparing the two diagnostic criteria, type I showed a worse prognosis compared to type II (type I mOS 84 days [CI 44.0; 124.0] versus type II mOS 198 days [CI 162.6; 233.4], *p* = 0.006, Fig. 2). No difference in response to treatment could be detected between the linear versus nodular MRI type (*p* = 0.717). There was also no difference in response to treatment when splitting type I and type II and the individual MRI subtypes (type I *p* = 0.371, type II *p* = 0.787). Furthermore, no difference in survival between the different diagnostic types depending on the application form of therapy (concomitant versus sequentially; type I *p* = 1.000, type II *p* = 0.751), and no differences in survival depending on the primary tumor type could be detected (breast *n* = 12, lung cancer *n* = 15, other *n* = 10, nonsolid *n* = 3; *p* = 0.479).

## Discussion

Toxicities of intrathecal chemotherapy in patients with leptomeningeal metastasis have previously been reported in the literature. Little, however, is known on the toxicity of combined intrathecal therapies with WBRT. This is one of the largest recently published retrospective studies reporting on the toxicity of intrathecal liposomal cytarabine combined with WBRT in patients with LM.

The discrimination of treatment-induced toxicity in our patient cohort was challenging especially when implementing a multimodal therapeutic approach. We tried to discriminate between intrathecal chemotherapy- and WBRT-induced toxicity using the NCI CTCAE and RTOG toxicity criteria. NCI CTCAE toxicity criteria covers chemotherapy-induced side effects [18], whereas RTOG classification considers radiogenic adverse events [17]. While these classifications broadly overlap, there are nevertheless clear differences. When it comes to dermatological side effects such as acute radiation dermatitis, RTOG toxicity criteria is better for categorizing local radiogenic side effects compared to NCI CTCAE toxicity criteria [20].

In our study, 25% of the patients experienced acute AEs grade 3 or higher according to RTOG toxicity criteria and 55% showed AEs ≥grade 3 according to NCI CTCAE toxicity criteria. Neurological complications (including headache, cognitive disturbances and cranial nerve dysfunction; around 20% of cases) were followed by hematological toxicities (10%). From the literature, neurological complications after intrathecal chemotherapy with liposomal cytarabine in patients with LM are similar and range between 16 and 50% [21–24]. In a retrospective case series of 120 patients treated with intrathecal liposomal cytarabine for LM, NCI CTCAE ≥grade 3 neurotoxicity was seen in 28 patients (23.3%). The most common toxicities included chemical meningitis (17.5% in ventricular administration versus 15% lumbar), conus medullaris/cauda equina syndrome (5%), decreased visual acuity (5% versus 2.5%), encephalopathy (5%), leukoencephalopathy (7.5% versus 2.5%), myelopathy (2.5%), radiculopathy (1.3% versus 5%) and seizures (1.3% versus 2.5%) [24]. When comparing intrathecal MTX and liposomal cytarabine in a randomized, controlled trial in patients with solid tumor neoplastic meningitis, treatment-related grade 3 toxicities were similar according to the Cancer and Leukemia Group B (CALGB)-expanded Common Toxicity Criteria [13]. In a prospective phase II clinical trial investigating concomitant intra-CSF MTX plus dexamethasone with focal radiotherapy for patients with LM from various solid tumors with adverse prognostic factors, 12/59 patients (20.3%) experienced grade 3–4 toxicities according to NCI CTCAE v3.0 criteria [14]. In our study the frequency of AEs of combined treatment was higher (55%). In contrast, we did not observe severe neurological AEs such as encephalopathy and radiculitis. Only 2 patients (10%) presented with acute drug-related arachnoiditis.

Overall, it is difficult to distinguish whether the severe neurological complications resulted from the performed WBRT or represented a cumulative toxicity from the multimodal treatment regime or have been an expression of the underlying progressive tumor disease. To ensure suitable clinical managements and strategies, signs and symptoms due to treatment related side effects should be discriminated from LM-induced neurological symptoms. As recommended by the Leptomeningeal Assessment in Neuro-Oncology (LANO) group, a comprehensive neurological examination using a standard evaluation form should be carried out at diagnosis and follow-up [15]. A broad knowledge of intrathecal chemotherapy-related and radiotherapy-induced toxicities can, however, aid to differentiate treatment-related symptoms from tumor progression. An intensified diagnostic procedure including MRI [25], recurrent CSF analysis [26] and additional F‑18 fluorodeoxyglucose (FDG) positron emission tomography/computed tomography (PET/CT) [27] can also be helpful to differentiate therapy-related side effects from disease progression. Nevertheless, the discrimination of treatment-induced toxicity in our patient cohort was challenging especially when implementing a multimodal therapeutic approach.

Interestingly, patients with response to treatment or stable disease more commonly experienced RTOG toxicities ≥grade 3. This might be due to the fact that six progressive patients prematurely terminated WBRT and therefore did not experience toxicities. Otherwise, there are several studies demonstrating an association with increased chemotherapy-induced toxicity and better efficacy regarding disease control and clinical outcome [28]. With regard to our patient cohort, it might appear that the occurrence of severe treatment-induced toxicity, defined as adverse events ≥grade 3 according to NCI CTCAE and RTOG toxicity criteria, leads to more efficacy and procrastination of LM.

In our study population, 31 patients were treated concomitantly and 9 patients sequentially. One might speculate that a concomitant treatment approach might result in a more efficient symptom control as well as stabilization and prevention of neurological deficits. Due to the unbalanced patient groups, the question on efficacy and difference in treatment toxicity could not be successfully answered as no differences in survival and toxicities between the two treatment approaches were detected.

Regarding to single treatment approaches, Glantz et al. could demonstrate that patients with solid tumor leptomeningeal metastasis who have been treated with DepoCyte® alone showed a median survival of 105 days [13]. A clinical trial conducted by Shaprio et al. shows a progression-free survival of 35 days when administered Cytarabine liposome injection alone in patients with leptomeningeal metastasis compared to 43 days when given combined methotrexate and non-liposomal cytarabine [29]. In our patient cohort, median overall survival was 124 days and median time to neurological progression was 52 days when conducting a multimodal treatment approach including DepoCyte® and WBRT. Hence, a combination of intrathecally given liposomal cytarabine and WBRT for the treatment of leptomeningeal metastasis shows an acceptable safety profile and may indicate a trend towards improved efficacy. However, in our opinion direct comparisons to other studies with respect to efficacy are highly problematic given the heterogenous patient populations.

In the second part of our study, we took the opportunity to evaluate EANO-ESMO criteria for diagnosis and response in LM patients receiving a combination treatment of intrathecal chemotherapy and WBRT. We found that diagnostic criteria as well as the response assessment were significantly associated with survival. None of the other factors evaluated (KPS score <60, median age, extensive systemic disease with extraneuronal metastases) were associated with survival in patients with LM [30–32].

Due to its limited penetration into solid tumor lesions [33], intra-CSF chemotherapy is often considered for patients with type 1 LM only [15]. In our study both types were treated by intrathecal chemotherapy and no differences in survival were detected. Recently, interesting data on the EANO-ESMO LM subtypes in a cohort of 245 patients were presented in an abstract at the SNO (Society of Neuro-Oncology) annual meeting 2020 [34]. The authors showed that, as in our cohort, patients with confirmed LM had inferior outcome compared to patients with probable or possible LM, and that type I patients had inferior outcome compared to type II patients. Nodular disease was a negative prognostic factor in type II LM, but not in type I LM. This finding was not reproducible in our study cohort, perhaps due to the smaller patient population. Most interestingly, as in our study, the EANO-ESMO response criteria were highly prognostic of survival and the authors concluded that these criteria should be considered for stratification and overall design of clinical trials in the future [34]. Our findings, although performed in a more selected patient cohort, definitely strengthen this statement.

## Conclusion

Combined treatment with intrathecal liposomal cytarabine and WBRT shows an acceptable safety profile and may indicate a trend towards improved efficacy. In addition, the suggested EANO-ESMO criteria for diagnosis and treatment response assessment in LM proved to be valid in our patient cohort.

## Supplementary Information

Supplemental Tables
