# Supplementary material for: Whole brain radiotherapy combined with intrathecal liposomal cytarabine for leptomeningeal metastasis—a safety analysis and validation of the EANO-ESMO classification
Source: Strahlenther Onkol. 2022 Mar 10;198(5):475–83. doi: 10.1007/s00066-022-01910-9 (PMC9038800; doi:10.1007/s00066-022-01910-9)
Supplement: Supplementary file 1 — Supplemental Tables [file 66_2022_1910_MOESM1_ESM.docx]

Supplement Table 1.

Toxicity and concomitant systemic chemotherapy

|  | **NCI CTCAE occurence** |  | **p-value** | **RTOG toxicity**  **occurence** |  | **p-value** |
| --- | --- | --- | --- | --- | --- | --- |
| **Systemic chemotherapy** | **no** | **yes** |  | **no** | **yes** |  |
| no | 6 | 16 | 0.509^a^ | 7 | 14 | 0.740^a^ |
| yes | 7 | 11 |  | 7 | 11 |  |

NCI CTCAE, National Cancer Institute Common Toxicity Criteria Adverse Events V5.0; RTOG, Radiation Therapy Oncology Group.

^a^Fisher’s exact test.

Supplement Table 2.

EANO-ESMO responses 8-12 weeks after start of therapy [[13](#_ENREF_13)]

| **EANO-ESMO response** | **overall (n=40)** | **concomitant (n=31)** | **sequential (n=9)** | **p-value** |
| --- | --- | --- | --- | --- |
| stable^1^ | 9 (22.5) | 7 (22.6) | 2 (22.2) | 0.412^a^ |
| response^1^ | 8 (20.0) | 7 (22.6) | 1 (11.1) |  |
| progression^1^ | 17 (42.5) | 11 (35.5) | 6 (66.7) |  |
| suspicion of progression^1^ | 6 (15.0) | 6 (19.4) | 0 (0) |  |

EANO-ESMO, European Association of Neurooncology – European Society for Medical Oncology; n, number.

^1^absolute number (percentage).

^a^Fisher’s exact test.
